# Study on the Influence of Geometric Characteristics of Grain Membranes on Permeability Properties in Porous Sandstone

**DOI:** 10.3390/membranes11080587

**Published:** 2021-07-31

**Authors:** Run Shi, Huaiguang Xiao, Chengmeng Shao, Mingzheng Huang, Lei He

**Affiliations:** 1 Nick Ceiling Pty. Ltd., St Peters, NSW 2044, Australia; aushirun@gmail.com; 2 School of Civil Engineering, Southeast University, Nanjing 211189, China; helei_civil@seu.edu.cn; 3 The 3rd Construction Company Ltd. of China 16th Corporation, Huzhou 313000, China; shaochengmeng.16g@crcc.cn; 4 Chang’an-Dublin International College of Transportation, Chang’an Univeristy, Xi’an 710018, China; mingzheng.huang@ucdconnect.ie

**Keywords:** porous sandstone, grain membranes, mapping method, lattice Boltzmann, permeability

## Abstract

Studying the influence of grain characteristics on fluid flow in complex porous rock is one of the most important premises to reveal the permeability mechanism. Previous studies have mainly investigated the fluid flow laws in complex rock structures using an uncontrollable one single parameter of natural rock models or oversimplified control group models. In order to solve these problems, this paper proposes a novel method to reconstruct models that can independently control one single parameter of rock grain membranes based on mapping and reverse-mapping ideas. The lattice Boltzmann method is used to analyze the influence of grain parameters (grain radius, space, roundness, orientation, and model resolution) on the permeability characteristics (porosity, connectivity, permeability, flow path, and flow velocity). Results show that the grain radius and space have highly positive and negative correlations with permeability properties. The effect of grain roundness and resolution on permeability properties shows a strong regularity, while grain orientation on permeability properties shows strong randomness. This study is of great significance to reveal the fluid flow laws of natural rock structures.

## 1. Introduction

Rock is a kind of complex geological body containing a large number of continuous and discontinuous structures. These discontinuous structures cause the penetrability of the rock at any scale, and they dominate the hydrological behavior of the rock. Therefore, the study of fluid flow laws in discontinuous structures has great significance for the prevention and control of inrush water in rock mass engineering [1] and the improvement of oil extraction [2] and gas extraction [3,4] efficiency. The discontinuous structures of the rock are extremely complex, so the structures should be quantitatively characterized, and then the fluid flow laws in the rock are possible to be revealed by analyzing the fluid behavior with different characterization parameters.

In order to characterize the discontinuous structures of the rock and obtain the fluid flow behaviors in the structures, a variety of research methods have been adopted. For the permeability experiments of the rock in the laboratory, computerized tomography (CT) can capture the discontinuous structure inside the rock [5], and the steady-state and non-steady-state methods can measure the rock permeability [6,7,8], and nuclear magnetic resonance (NRM) can capture the fluid flow process to some extent [9]. The pore structure model and permeability obtained by these methods are realistic. However, the fluid flow paths in rock structures are challenging to catch with real-time and high fidelity. Furthermore, due to the random structure of natural rock, the quantitative characterization parameters between different rocks do not have regularity, so the permeability laws of rock structure are hard to study. To reveal the fluid flow paths, the theoretical analysis method for rock permeability can realize quantitative characterization parameters between different rock models with a single variable [10]. It can also obtain permeability results and visual flow paths that can be used to analyze the influence of rock structural parameters on permeability behavior. However, this method can only solve the permeability behaviors of simplified models and cannot solve the nonlinear permeability problem of complex rock structures. With the rapid development of high-performance computing platforms and numerical algorithms, the numerical simulation method has been widely used in permeability analysis of complex structures in recent years. This method has the advantages of solving complex problems, strong visualization, and low cost.

Constructing a numerical model that can simulate the natural rock structures (real control group) and control the independent change of characterization parameters (single control variable) is vital to study the fluid flow laws in complex rock structures based on the numerical simulation method. The modeling of pore structures is usually divided into direct and indirect methods. The direct methods refer to obtain two-dimensional or three-dimensional natural rock pore structures using physical equipment (such as the camera, CT) and image processing technology [11]. The model established by this method can reproduce the structural characteristics of the natural rock (real control group), and the permeability behavior of the natural rock can be obtained with the support of scientific CFD algorithms. However, due to the random differences between different rocks, the characterization parameters are intermingled with each other, which causes the hard to realize independent variations of the characterization parameters. The indirect method refers to using random distribution to reproduce some parameter characterizations in a new model. Parameterization methods mainly include the fractal method [12], the Gaussian field method [13], the simulated annealing method [14], the QSGS parameter method [15], and the Markov chain Monte Carlo method [16]. These methods can establish plausible pore models and control the single variation of characteristic parameters, but the complementary phase (grain structure) of pore structures is difficult to reflect the morphology of natural grains, which cannot form a real control group. Xiao et al. [17,18] used digital twinning technology and texture synthesis method to build highly realistic rock moso-structures to keep the pore and grain structures natural and realistic. However, these methods cannot achieve single and independent changes in characterization parameters.

Due to the high complexity and randomness of pore structures, it is challenging to describe the pore structures accurately. The boundary (membranes) of mineral grains delineates the pore structures over time by geological processes. In other words, the pore structures that are structurally complementary to the grains are the product of the interaction between mineral grains. Therefore, if the grain structures of the rock can be accurately described, the pore structures will also be accurately described. Compared with the complex pore structures, the relatively regular grain structures can reveal the fluid flow laws of porous rock more easily. Many scholars have studied the permeability behaviors by changing the space [19], radius [10], and roundness [20] of grains and obtained helpful conclusions. However, the control groups in this research were simplified rock models, and they could not accurately reflect the actual permeability behaviors. In this case, the models with various parameters derived from these simplified control groups are likely to reflect non-accurate permeability laws. Although the above studies can control the single variable of the representational parameters, the results of the control groups are not convincing.

With regard to permeability simulation, the finite volume method, finite difference method, and finite element method, based on the Navier–Stokes (NS) equations, are mainstream algorithms in most commercial software. However, the NS-based methods for solving the fluid flow problems in complex porous media are challenging because the mesh of these methods is difficult to guarantee both model and computational reliability. By contrast, the lattice Boltzmann method (LBM) is based on the pixel (lattice) scale, which can reproduce complex pore structures and give accurate computational fluid results. The advantages of LBM in complex porous media have been applied in many studies, such as heat transfer [21,22] and water seepage [17].

This paper aims to study the influence of geometry characteristics of grains on fluid flow laws by changing the geometric parameters of grains in natural rocks based on natural rocks as the control group and the controlling single variable method. The two-dimensional image of the natural pore sandstone provides an actual control group, and the discrete mapping and reverse-mapping ideas are used to achieve single and controllable grain parameters. Furthermore, the lattice Boltzmann method is used to reveal the fluid flow laws of the pore sandstone model under different characterization parameters.

## 2. Materials and Methods

### 2.1. Materials

**Figure 1:**
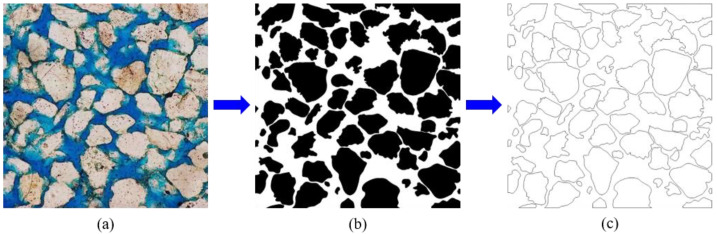
The process for obtaining grain boundaries (membranes) of porous sandstone. (**a**) Porous sandstone; (**b**) Grain separation; (**c**) Grain boundaries.

The model in this research is from Oklahoma’s Permian-age Sandstone [23]. As shown in Figure 1a, the physical size of the porous sandstone is approximate to 1 mm × 1 mm, in which the mineral grains are off-white with a diameter of about 0.2 mm, and the pores are dyed blue. In order to study the influence of mineral grains in this porous sandstone on permeability behavior, the manual delineate method was used to separate the 71 grains completely, as shown in Figure 1b. Then, image processing technology was used to identify the mineral grain boundary and obtain the skeleton structure of the boundary, as shown in Figure 1c. The area, equivalent radius, perimeter, and roundness of separated mineral grains are shown in Table 1.

### 2.2. Mapping and Reverse-Mapping Method

**Figure 2:**
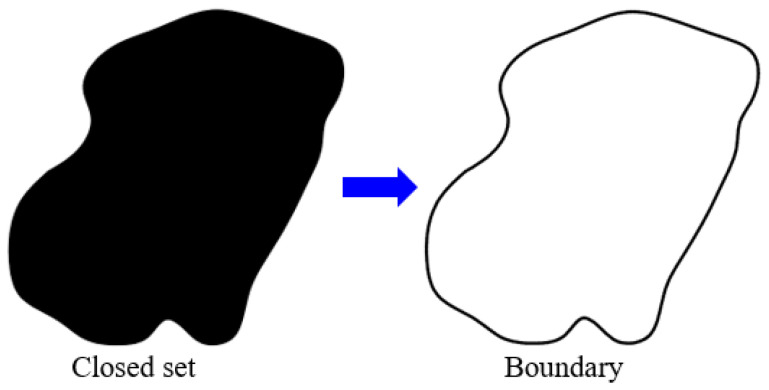
Dimension reduction of grain topologically closed sets.

Each grain in the porous sandstone is considered intact and homogeneous, and each grain model can be abstracted as a topologically closed set (as shown in Figure 2), which is a two-dimensional infinite point set in Euclidean space. There is much redundancy in describing topologically closed sets using two-dimensional coordinates in Euclidean space. Combined with the dimensionality reduction idea of manifolds, topologically closed sets can be accurately described using boundaries, as shown in Figure 2. In other words, if the grain boundary is accurately described, the intact grain will also be accurately described. The grain boundary in natural rocks is characterized by high complexity and randomness, so it is not easy to characterize the grain boundary with geometric parameters accurately. Substitutively, the discrete and high-density points is an effective method to describe complex boundary structure.

**Figure 3:**
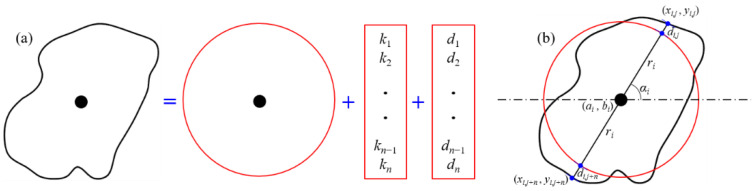
The mapping and reserve-mapping principle. (**a**) The mapping and reserve-mapping process between irregular boundary and circle boundary; (**b**) Parameters relationships between irregular boundary and circle boundary.

In order to study the geometric characteristics of the grains (such as size, position, roundness, and orientation), parameterized analysis of the grain characteristics is required. The radius of an equivalent circle, the centroid, the distance between grain boundary and the equivalent circle, the angle between the long axis and horizontal line represent the size, the position, the roundness, and orientation, respectively. To describe the irregular boundary of the grain, discrete points of the boundary are mapped onto the circle. All discrete points on the boundary can be recorded only by recording two sets of parameters (slope of the centroid and boundary point, mapping distance between the circle and boundary) under the condition of constant centroid and radius, as shown in Figure 3a. Thus, an unique boundary point can be determined by given an endpoint of a line segment (centroid), a slope, and a signed distance (radius and mapping distance). The original model can be perfectly reserve-mapping with two sets of parameters (slope and mapping distance). As shown in Figure 3b, the radius, centroid position, mapping distance, and slope can be adjusted in the reserve-mapping process to change the size, spacing, roundness, and orientation parameters of grains, respectively, by using Formulas (1)–(4)

(1) $$k_{i}=tan\alpha_{i}=\frac{y_{i,j}-a_{i}}{x_{i,j}-b_{i}}$$

(2) $$d_{i}=\pm \sqrt{{\left(y_{i,j}-a_{i}\right)}^{2}+{\left(x_{i,j}-b_{i}\right)}^{2}}-r_{i}$$

(3) $$x_{i,j}=a_{i}+sign\left(a_{i}-x_{i,j}\right)\times \frac{r_{i}+d_{i}}{\sqrt{{tan}^{2}\alpha_{i}+1}}$$

(4) $$y_{i,j}=b_{i}+sign\left(a_{i}-x_{i,j}\right)\times tan^{2}\alpha \times \frac{r_{i}+d_{i}}{\sqrt{{tan}^{2}\alpha_{i}+1}}$$

(*a_i_,b_i_*)—grain centroid position; *r_i_*—grain equivalent radius; *d_i_*—grain mapping distance; *α_i_*—grain orientation; (*x_i,j_, y_i,j_*)—the *j*-th point on the boundary of the *i*-th grain.

#### 2.2.1. Size

**Figure 4:**
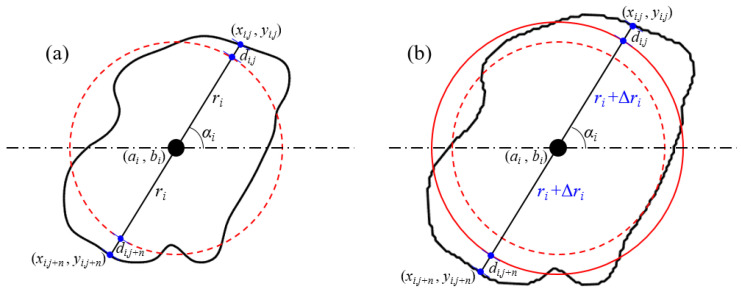
The principle of grain size change. (**a**) Original relationship between grain boundary and mapping circle. (**b**) The relationship between them with the grain radius increasing Δ*r_i_*.

Under the condition that the grain centroid coordinates, mapping distance, and slope remain unchanged, a single variation in grain size can be achieved by adjusting the radius, as shown in Figure 4. Assume that the grain radius increases by Δ*r_i_*, then the grain radius after the change is

(5) $$r_{i}^{\prime }=r_{i}+\Delta r_{i}$$

**Figure 5:**
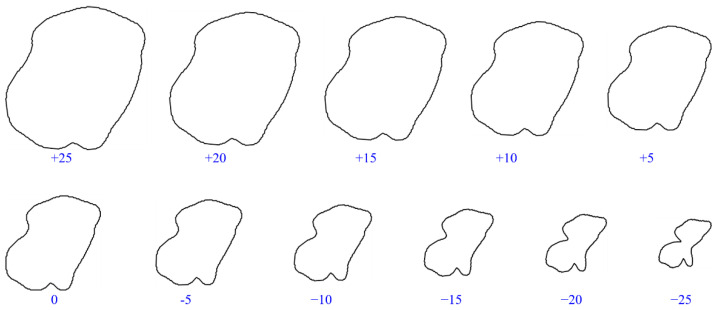
The results of grain size change.

The results of grain size change is shown in Figure 5.

#### 2.2.2. Position

**Figure 6:**
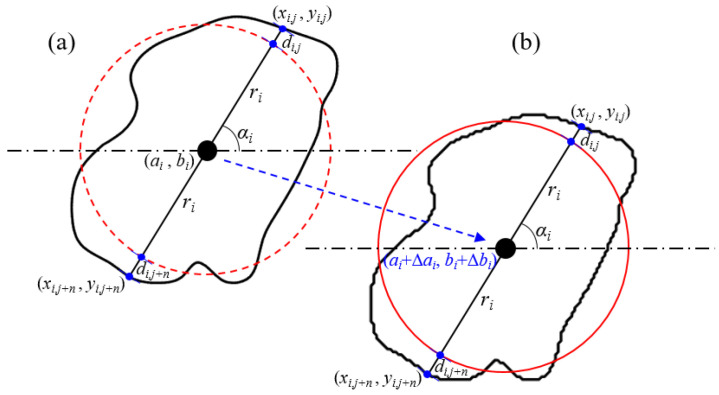
The principle of grain position change. (**a**) Original relationship between grain boundary and mapping circle. (**b**) The relationship between them with the coordinates increasing Δ*a_i_* and Δ*b_i_*.

Under the condition that grain radius, mapping distance, and slope remain unchanged, the only change of grain position and spacing can be realized by adjusting centroid position, as shown in Figure 6. Assume that Δ*a_i_* and Δ*b_i_* are added in the horizontal and vertical coordinates of the grain centroid, respectively, then the changed grain position is

(6) $$\left(a_{i}^{\prime },\;b_{i}^{\prime }\right)=\left(a_{i}+\Delta a_{i},\;b_{i}+\Delta b_{i}\right)$$

**Figure 7:**
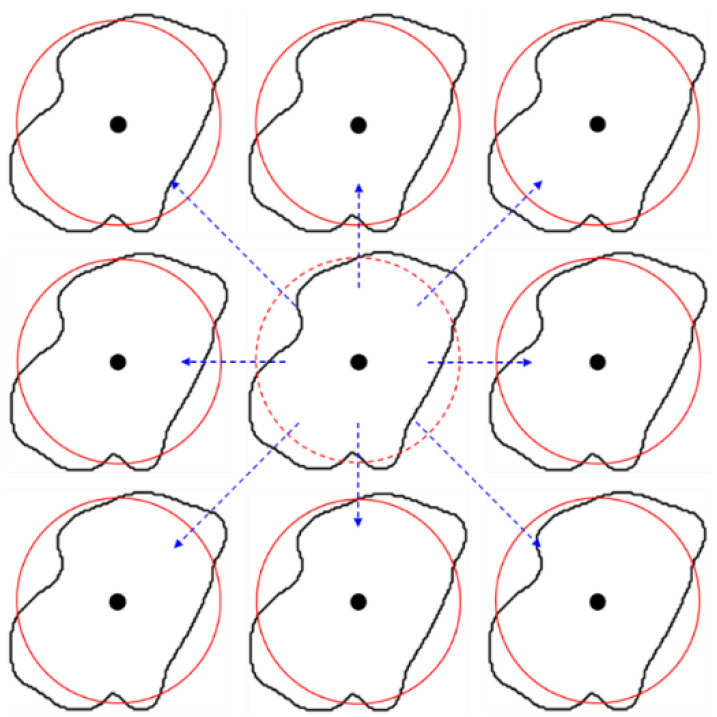
The results of the grain position change.

The results after the change of grain position is shown in Figure 7.

#### 2.2.3. Roundness

**Figure 8:**
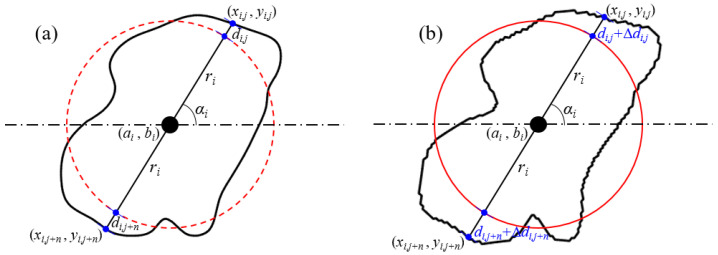
The principle of grain roundness change. (**a**) Original relationship between grain boundary and mapping circle. (**b**) The relationship between them with the mapping distance increasing Δ*d_ij_*.

Under the condition that the centroid coordinates, radius, and slope of the grain remain unchanged, a single change in the roundness of the grain can be achieved by adjusting the mapping distance, as shown in Figure 8. Assuming that the mapping distance increases Δ*d_ij_*, then the mapping distance after the change is

(7) $$d_{ij}^{\prime }=d_{ij}+\Delta d_{ij}$$

**Figure 9:**
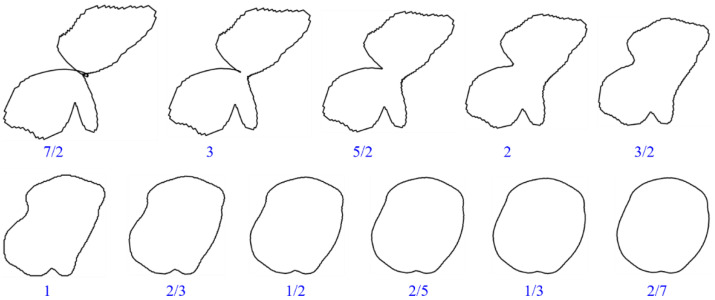
The results of grain roundness change.

The result after changing the mapping distance of grains is shown in Figure 9.

#### 2.2.4. Orientation

**Figure 10:**
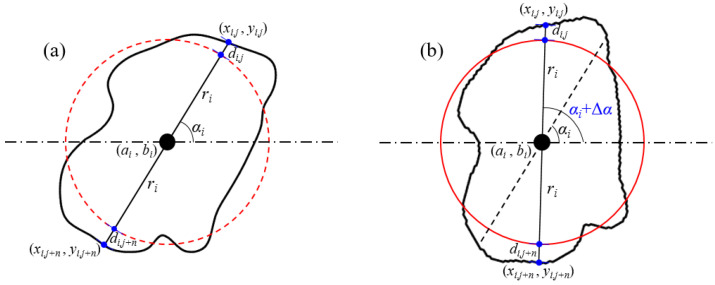
The principle of grain orientation change. (**a**) Original relationship between grain boundary and mapping circle. (**b**) The relationship between them under a Δα*_i_* counterclockwise rotation.

Under the condition that the centroid coordinates, radius, and mapping distance of grains remain unchanged, the single control variable grain orientation can be achieved by adjusting the slope of grains, as shown in Figure 10. Suppose the grain orientation rotates Δα*_i_* counterclockwise, then the slope of the point on the grain boundary after rotation is

(8) $$k_{i}^{\prime }=tan(\alpha_{i}+\Delta \alpha_{i})=\frac{tan\alpha_{i}+tan\Delta \alpha_{i}}{1-tan\alpha_{i}tan\Delta \alpha_{i}}=\frac{k_{i}+tan\Delta \alpha_{i}}{1-k_{i}tan\Delta \alpha_{i}}$$

**Figure 11:**
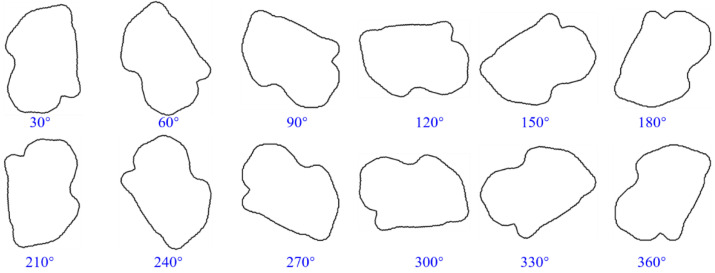
The results of grain orientation change.

The results of grain orientation changes are shown in Figure 11.

### 2.3. Lattice Boltzmann Method

The lattice Boltzmann method is a computational fluid dynamics method based on the kinetic theory. Compared with other CFD methods, it is good at solving the fluid flow process in complex structures. In this paper, the classical BGK solution model is adopted, and the collision and streaming rules between grains are used to calculate fluid seepage rules. The evolution equation as follows

(9) $$f\left(x+e\delta t,e,t+\delta t\right)-f\left(x,e,t\right)=-\frac{1}{\tau }\left[f\left(x,e,t\right)-f^{eq}\left(x,e,t\right)\right]$$

where *f* is a function of variables *t*, *x*, *e*, and *f(t, x, e)* represents the density distribution of grains which velocities are *ξ* at the position *x* at time *t*; *τ* refers to the average time interval between two collisions, also known as slack time or relaxation time; is the equilibrium distribution function.

In order to solve the Boltzmann–BGK (Bhatnagar–Gross–Krook) equation by discretizing the evolution equation, the Taylor expansion equation of Maxwell’s equilibrium state distribution function is

(10) $$f_{i}^{eq}=w_{i}\rho \left[1+\frac{3(e_{i}\cdot u)}{c^{2}}+\frac{9{\left(e_{i}\cdot u\right)}^{2}}{2c^{4}}-\frac{3u^{2}}{2c^{2}}\right]$$

where *ω_i_* is the weight coefficient; ρ is the density; *e_i_* is the discrete velocity; *u* is the velocity, and *c* is the lattice velocity.

**Figure 12:**
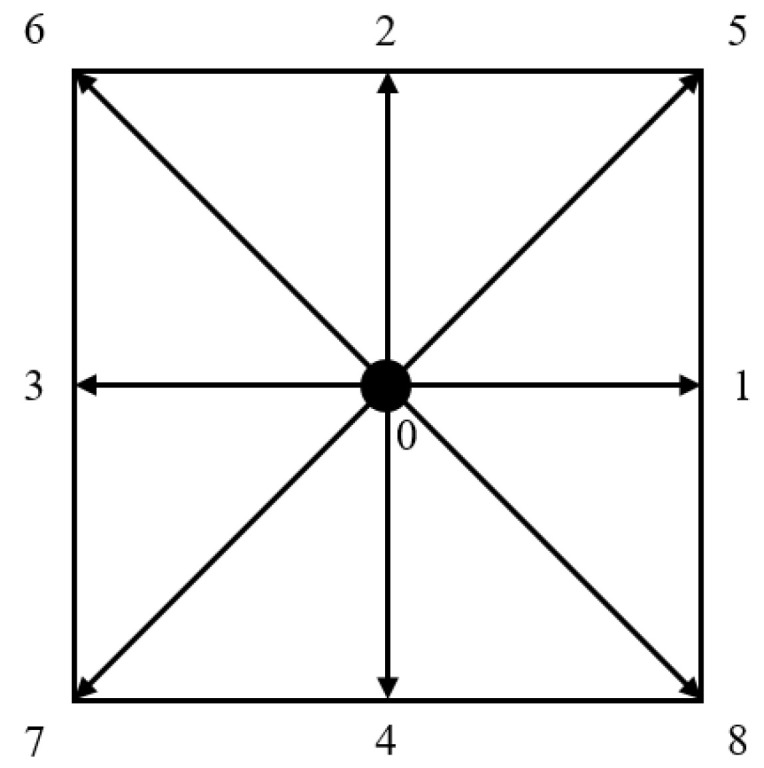
D2Q9 model.

In this research, the phase space discrete model of D2Q9 is adopted, as shown in Figure 12. According to the D2Q9 model, the discrete velocity set and weight coefficient of **e** are respectively calculated as follows:

(11) $$e_{i}=c\left(\begin{array}{l} 0\;1\;0\;-1\;\;\;0\;\;\;1\;-1\;-1\;\;\;\;1\\ 0\;0\;0\;\;\;\;0\;\;-1\;1\;\;\;\;1\;\;\;-1\;-1 \end{array}\right)$$

(12) $$w_{i}=\left\{ \begin{array}{l} 4/9,\;\;e_{i}^{2}=0\\ 1/9,\;\;e_{i}^{2}=c^{2}\\ 1/36,\;e_{i}^{2}=2c^{2} \end{array}\right.$$

Based on the Chapman–Enskog expansion formula, the macroscopic fluid density *ρ*, velocity **u**, pressure *p*, and viscosity coefficient *v* can be derived through the following equations:

(13) $$\rho =\sum_{i}f_{i}$$

(14) $$u=\sum_{i}f_{i}e_{i}/\rho$$

(15) $$p=c_{s}^{2}\rho \;\;\;\;\;$$

(16) $$v=\frac{c^{2}}{3}\left(\tau -0.5\right)\delta$$

The standard rebound boundary is adopted in this research. The boundary of the model is left in and right out, and the other boundaries are closed.

## 3. Results

In this section, the above methods are used to reconstruct the models of porous sandstone, and the permeability laws affected by the parameters of grain membranes are analyzed based on the reconstructed models. The simulation parameters in this paper are derived from the literature [17], which has been verified by the physical experiments.

### 3.1. Grain Radius

**Figure 13:**
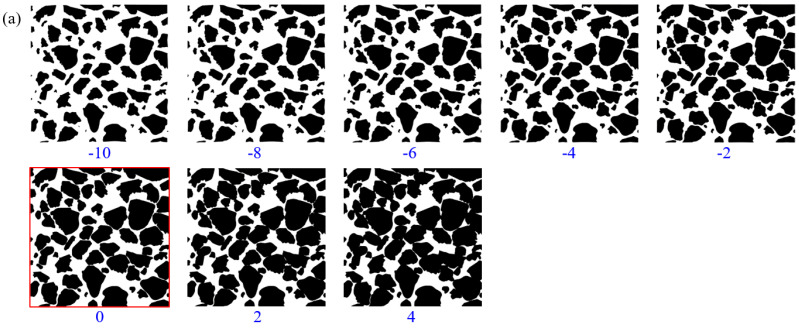

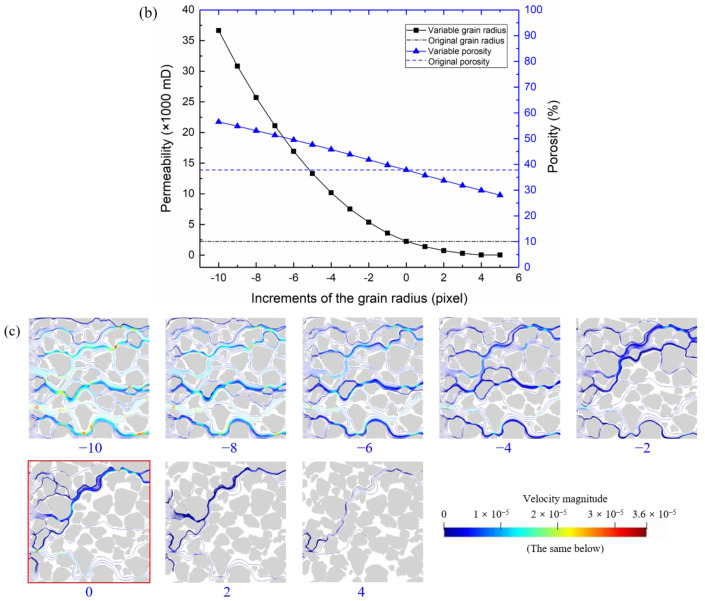
Influence of grain radius on permeability. (**a**) Pore models with different grain radiuses; (**b**) porosity and permeability of pore models with different grain radiuses; (**c**) flow paths and velocity of pore models with different grain radiuses.

Grain radius determines grain sizes, which affects the structure and connectivity of the pores surrounded by the grain membrane. In order to study the influence of membrane structure radius on pore structure characteristics, the equivalent grain radius in the original image (red box line) is reduced (−10, −8, −6, −4, and −2 pixels) and increased (2 and 4 pixels) under the condition that the model size (700 × 700 pixels) remain constant, as shown in Figure 13a. When the equivalent grain radius is small, the pore channels formed by grain membranes have large sizes and good connectivity. With the increase of the equivalent grain radius, the distance between grains gradually reduces, and then contact or overlap, making the channels formed by the grain membranes become narrow, and the connectivity decreases gradually.

In order to study the effect of the pore structure changed by the equivalent grain radius on the permeability characteristics, the permeability, and flow path under different grain radii were analyzed, respectively. As shown in Figure 13b, with the increase of equivalent grain radius, permeability presents a decrease in an approximate power function type. The permeability of the original model was 2228 mD. When the equivalent grain radius was reduced by 10 pixels, the permeability of the model was 36,636 mD, which is exceptionally high. When the equivalent grain radius was increased by 5 pixels, the permeability of the model was 15 mD, which is extremely low. As shown in Figure 13c, when the equivalent grain radius was small, the number of fluid flow channels from left to right is large, and the flow velocity was high. With the increase of the equivalent grain radius, both the number of channels and flow velocity decrease gradually. When the equivalent grain radius was increased by 4 pixels, only one narrow flow channel and the flow velocity was very low.

### 3.2. Grain Space

**Figure 14:**
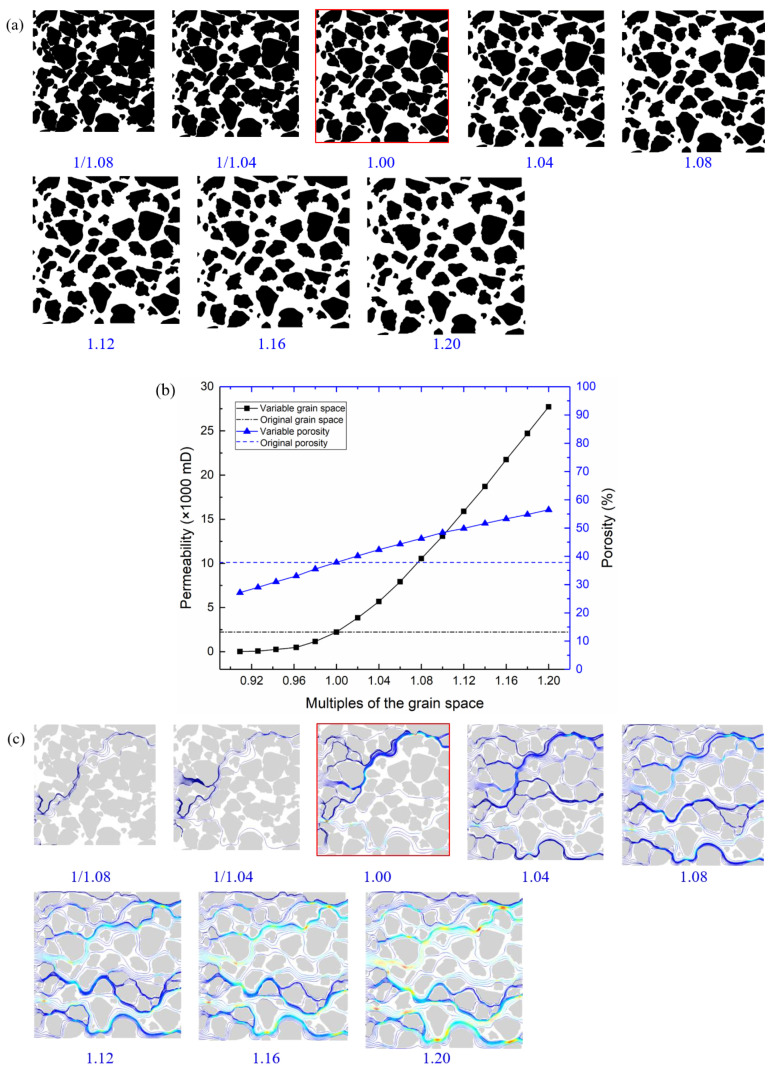
Influence of grain space on permeability. (**a**) Pore models with different grain spaces; (**b**) porosity and permeability of pore models with different grain spaces; (**c**) flow paths and velocity of pore models with different grain spaces.

In porous sandstones, pore media is usually filled between mineral grains, so the larger the space between the grains, the greater the pore media will be. Under the condition that the size and shape of the grains are constant, increasing grain space, the model size and spacing are raised by the same multiple. The length of the original image (red box line) is 700 pixels while adjusting the space by 1/1.08, 1/1.04, 1.04, 1.08, 1.12, 1.16, and 1.20 times and the corresponding model lengths are 648, 673, 728, 756, 784, 812, and 840 pixels, respectively. As shown in Figure 14a, when the grain space of the original model is reduced, the grains contact and overlap, which have similar characteristics to that of increasing the grain radius. With the increasing grain space, the pore channels between grains become wide, and the connectivity becomes well.

As shown in Figure 14b, the permeability of the original model is 2228 mD. When the grain space reduces, the permeability gradually decreases until it reaches zero. The permeability increases linearly with the rise of grain space. When the spacing is increased by 1.2 times, the permeability reaches 27,723 mD. As shown in Figure 14c, the flow channel and velocity are tiny when the spacing is minimal. With increasing grain spacing, the flow channel and velocity increase.

### 3.3. Grain Roundness

**Figure 15:**
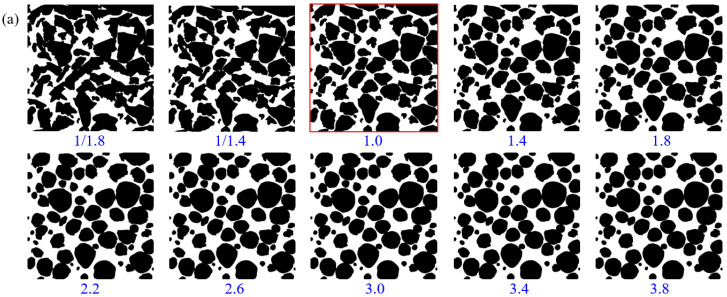

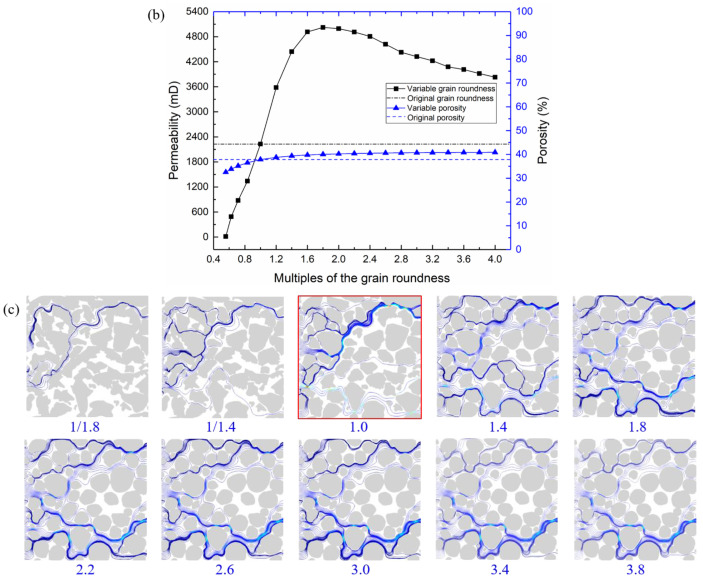
Influence of grain roundness on permeability. (**a**) Pore models with different grain roundness; (**b**) porosity and permeability of pore models with different grain roundness; (**c**) flow paths and velocity of pore models with different grain roundness.

Mineral grains of natural rocks are irregular, and their irregularity largely determines the shape of the membrane between solid and liquid, thus affecting the permeability characteristics. The grain roundness of the original model (red box line) is extremely diverse, ranging from approximate round grain shapes to very complex shapes. In order to study the influence of grain roundness on permeability characteristics, all grains in the rock model are reduced or increased by changing the mapping distance on the equivalent circle under the condition that the model size remains unchanged, as shown in Figure 15a. When the roundness is tiny, the grain complexity is high with a long major-axis and a short minor-axis. The increase of the major axis will make the grains contact or overlap and reduce the connectivity of the pores. As the roundness of grains increases, the major-axis gradually decreases while the minor-axis gradually increases, and the shape of grains becomes more and more round. Thus, pore connectivity and porosity show a slow increase and then stabilize, as shown in Figure 15b.

When the grain roundness is small, the permeability is low, as shown in Figure 15b. With the increase of roundness, permeability increases rapidly at first and then decreases slowly. When the roundness is 1.8 times of the original model, the maximum permeability reaches 5022 mD. As shown in Figure 15c, the flow channel and velocity increased rapidly and then decreased slowly with the increase of roundness.

### 3.4. Grain Orientation

**Figure 16:**
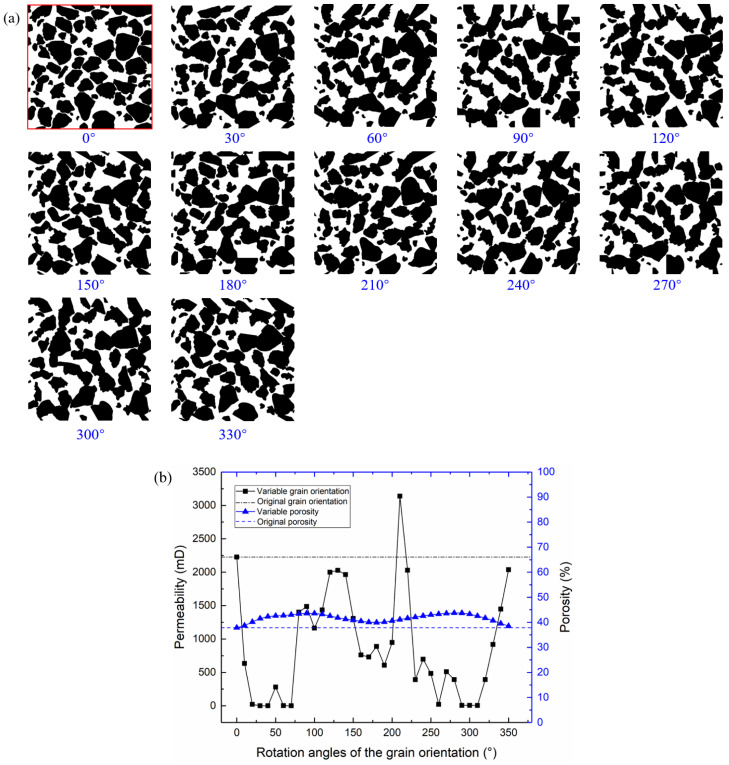

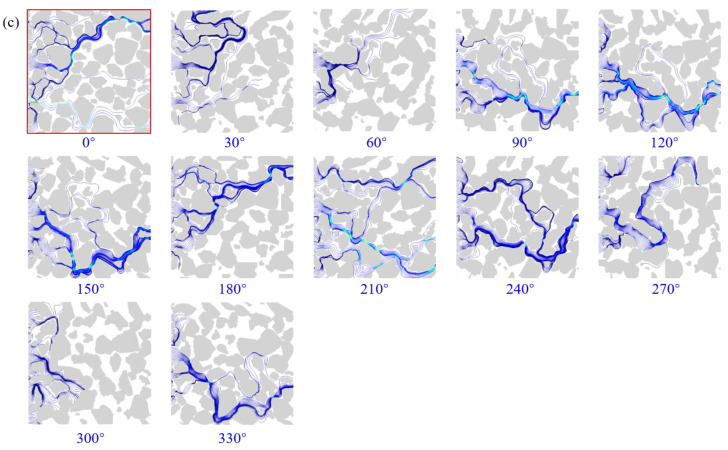
Influence of grain orientation on permeability. (**a**) Pore models with different grain orientations; (**b**) porosity and permeability of pore models with different grain orientations; (**c**) flow paths and velocity of pore models with different grain orientations.

Grains in natural rocks have diverse orientations, and grain orientation mainly affects the contact relationship between adjacent grains, including separation, tangency, or intersection. In order to reveal the influence of grain orientation on permeability characteristics, different orientated grain models are obtained by rotated counterclockwise at the same angle of all grains under the condition that the model size, grain size, and shape remained unchanged, as shown in Figure 16a. The change of grain orientation alters the pore structure and connectivity formed between adjacent grains and then affects the pore structure characteristics of the whole model. As shown in Figure 16b, the rotation angle of grain orientation has little influence on the porosity. The difference between the maximum and minimum porosity is only 5.75%. When the rotation angle of grain orientation is between 0–180°, the porosity presents a slow increase and then slowly decrease, and the porosity change law between 180° and 350° shows a similar tendency to that between 0°and 180°.

As shown in Figure 16b, with the change of grain orientation rotation angle (every 10°), there is no strong regularity between permeability and rotation angle. Only at an angle of 210°, was the model permeability (3139 mD) larger than the original permeability. The model permeability at some angles was almost zero (such as 20°, 30°, 40°, 60°, 70°, 260°, 290°, 300°, and 310°). At other angles, the permeability fluctuated randomly between 0 and 2228 mD (the permeability of the original model). The effect of grain orientation on permeability had strong randomness. As shown in Figure 16c, with the change of grain rotation angle, the flow channel and velocity have a large random change. The fluid in the model with very low permeability could not flow from the left to the right side of the model.

### 3.5. Model Resolution

**Figure 17:**
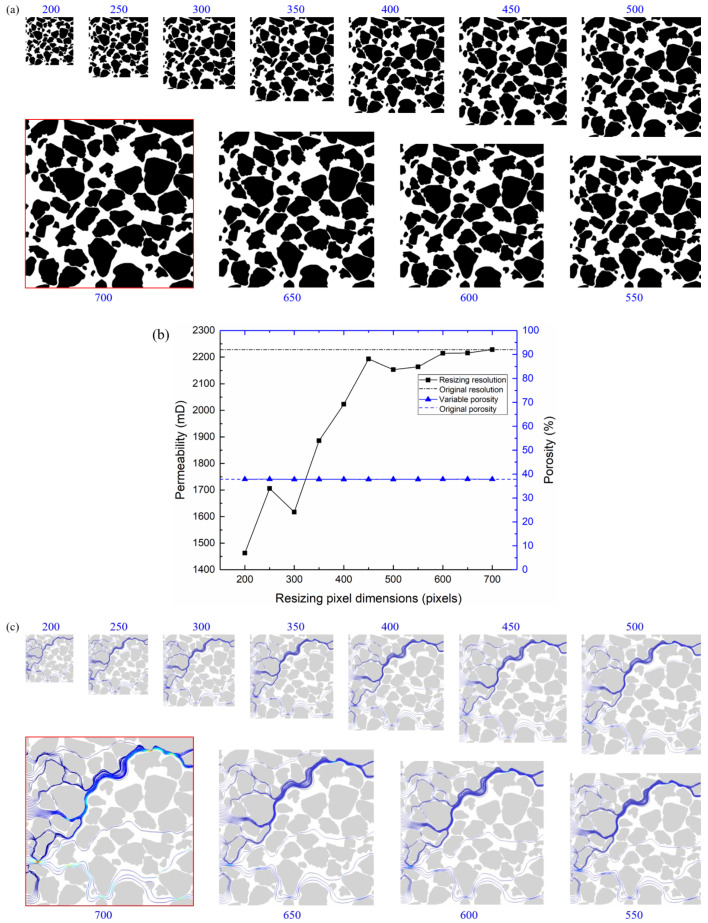
Influence of model resolution on permeability. (**a**) Pore models with different resolution; (**b**) porosity and permeability of pore models with different resolution; (**c**) flow paths and velocity of pore models with different resolution.

The lattice Boltzmann method, based on image pixel, was used to calculate the permeability of pore models. A pixel is a relative scale, which can represent different physical dimensions. To analyze the influence of image models under different resolutions on permeability characteristics, this study obtained models with the side length of 200, 250, 300, 350, 400, 450, 500, 550, 600, and 650 pixels, as shown in Figure 17a. The results show the pore structures between grains only change with the resolution, and its shape did not change, so the change of model resolution did not affect the porosity, as shown in Figure 17b.

With the increase of model resolution, the permeability gradually increased and then became stable, as shown in Figure 17b. When the model side length was 200 pixels, the minimum permeability was 1463 mD. When the model side length was 450 pixels, the permeability was 2193 mD, similar to the 2228 mD at 700 pixels. As shown in Figure 17c, the change of model resolution had little influence on the seepage path, but the seepage velocity gradually increases when the resolution is higher.

## 4. Discussion

Based on the actual control group of porous sandstone, this paper constructed models that can describe the single parameter variance of grains by mapping and reverse-mapping ideas and by further analyzing the influence of each parameter on the permeability characteristics of pore structure. Basing on actual structure with one single parameter as an influencing factor to analyze the permeability characteristic is of great significance to reveal the permeability mechanism of complex structures.

In this paper, the actual sandstone model is used as the control group. Based on the control single variable principle, the influence of grain radius, space, roundness, orientation, and model resolution on connectivity, porosity, permeability, flow path, and flow velocity. The influences of both grain parameters and the whole model on permeability characteristics are obtained. Compared with the simplified model as the control group, the actual control group ensures the accuracy of permeability characteristics under complex structures. Compared with the analysis of permeability characteristics under multiple groups of actual rock models, the principle of single-variable control ensures the independent regularity between parameter changes and permeability characteristics.

This paper reveals the influence of grain boundary parameters on permeability characteristics on the actual model, but it only shows the laws of changing a single parameter model. In contrast, multiple parameters changing simultaneously are more likely to leads to the difference of natural rocks. Besides, this paper mainly focuses on the two-dimensional model to study the influence of grain parameters on permeability characteristics. However, natural porous rocks exist in a more complex three-dimensional space, and the influence laws of parameters still need to be further verified in three-dimensional models.

Due to the limitations of this study and natural rock characteristics, the study of grain parameter sensitivity of three-dimensional porous rock will explain the permeability laws in natural rocks more truly. In the future, studying the influence of multiple parameter combinations on the seepage characteristics is of great significance to reveal the permeability mechanism under complex porous media.

## 5. Conclusions

This paper reconstructs models that can describe a single parameter change of grain boundary based on the actual sandstone. Combined with mapping and reverse-mapping ideas, the actual control group and the control of a single variable scientific method were used to analyze the model parameters’ influence on permeability characteristics. Firstly, with the increment of the grain radius from −10 to 5, the porosity and permeability decreased from 56.51 to 28.06%, from 36,635.51 mD to 14.85 mD, respectively. Secondly, with the multiples of the grain radius from 1/1.08 to 1.20, the porosity and permeability increased from 27.13 to 56.49%, from 11.67 mD to 27,723.21 mD, respectively. Thirdly, with the multiples of the grain roundness from 1/1.8 to 4.0, the porosity and permeability increased from 32.55 to 40.87%, from 13.20 mD to 3828.57 mD, respectively. Fourthly, the grain orientation change has less effect on porosity between 37.85 and 43.73%, while it had a strongly random impact on permeability between 0 to 3138.61 mD. Fifthly, with the model resolution increased, the porosity remained unchanged at 37.85%, while permeability shows increased trends and then stabilized from 1462.87 mD to 2228.25 mD.

In conclusion, different grain parameters have significant differences in the influence on permeability characteristics. From the perspective of grain radius, space, and roundness, there is a positive or negative correlation between porosity and permeability. However, there was almost no correlation between porosity and permeability in terms of grain orientation and resolution parameters. The change of model connectivity under different grain parameters directly affects the change of seepage paths. These conclusions can provide a reference for revealing the permeability law between different complex rock structures.

## Figures and Tables

**Table 1:** Grain geometry parameters (sorted by area from large to small).

No.	Area /Pixels	Equivalent Radius /Pixel	Perimeter /Pixel	Roundness	No.	Area /Pixels	Equivalent Radius /Pixel	Perimeter /Pixel	Roundness
1	16,089	71.56	499.92	0.77	37	3246	32.15	219.75	0.76
2	13,745	66.14	476.77	0.70	38	3219	32.01	252.89	0.59
3	11,868	61.46	446.09	0.69	39	2664	29.12	245.94	0.55
4	11,452	60.38	422.87	0.75	40	2573	28.62	210.94	0.67
5	11,266	59.88	428.02	0.72	41	2418	27.74	193.40	0.74
6	10,784	58.59	621.10	0.35	42	2312	27.13	235.47	0.49
7	9309	54.44	396.04	0.71	43	2064	25.63	234.90	0.45
8	9080	53.76	430.48	0.59	44	2009	25.29	188.76	0.66
9	7983	50.41	484.83	0.41	45	1842	24.21	230.67	0.40
10	7930	50.24	370.31	0.72	46	1837	24.18	160.17	0.83
11	7320	48.27	343.00	0.72	47	1734	23.49	168.89	0.70
12	7146	47.69	339.35	0.73	48	1710	23.33	166.04	0.73
13	6693	46.16	373.26	0.55	49	1681	23.13	208.37	0.45
14	6637	45.96	400.66	0.49	50	1545	22.18	176.45	0.57
15	6498	45.48	332.51	0.68	51	1354	20.76	148.44	0.72
16	6397	45.13	362.81	0.57	52	1188	19.44	133.53	0.78
17	6204	44.44	317.68	0.70	53	975	17.62	114.47	0.85
18	6035	43.83	331.56	0.67	54	964	17.52	132.79	0.69
19	5975	43.61	338.20	0.62	55	949	17.38	136.12	0.59
20	5961	43.56	334.25	0.63	56	891	16.84	129.42	0.63
21	5671	42.49	296.29	0.74	57	816	16.12	107.51	0.80
22	5175	40.58	348.43	0.50	58	740	15.35	102.46	0.80
23	5079	40.21	349.42	0.49	59	682	14.73	144.48	0.38
24	5060	40.13	281.61	0.74	60	626	14.12	98.63	0.78
25	5013	39.94	288.02	0.69	61	563	13.39	120.57	0.47
26	4881	39.42	277.90	0.74	62	526	12.94	124.82	0.39
27	4497	37.83	267.34	0.73	63	521	12.88	89.63	0.74
28	4242	36.75	266.25	0.70	64	521	12.87	89.75	0.74
29	4216	36.63	288.08	0.61	65	498	12.59	104.58	0.53
30	4127	36.24	279.46	0.62	66	406	11.37	72.70	0.88
31	4054	35.92	278.28	0.62	67	268	9.24	64.43	0.76
32	3944	35.43	265.95	0.63	68	252	8.95	63.38	0.74
33	3885	35.16	291.08	0.52	69	243	8.80	54.94	0.91
34	3647	34.07	252.40	0.72	70	156	7.06	56.72	0.63
35	3506	33.41	231.89	0.74	71	144	6.76	46.05	0.79
36	3295	32.38	303.86	0.43					

## Data Availability

Not applicable.
